# Supplementary material for: Splice-Junction-Based Mapping of Alternative Isoforms in the Human Proteome
Source: Cell Rep. Author manuscript; Available in PMC 2020 Jan 15. (PMC6961840; doi:10.1016/j.celrep.2019.11.026)
Supplement: 2 [file NIHMS1546469-supplement-2.zip › DF1/PXD010154/Heart/Heart_73_TTN_APHVEFLRPL.pdf]

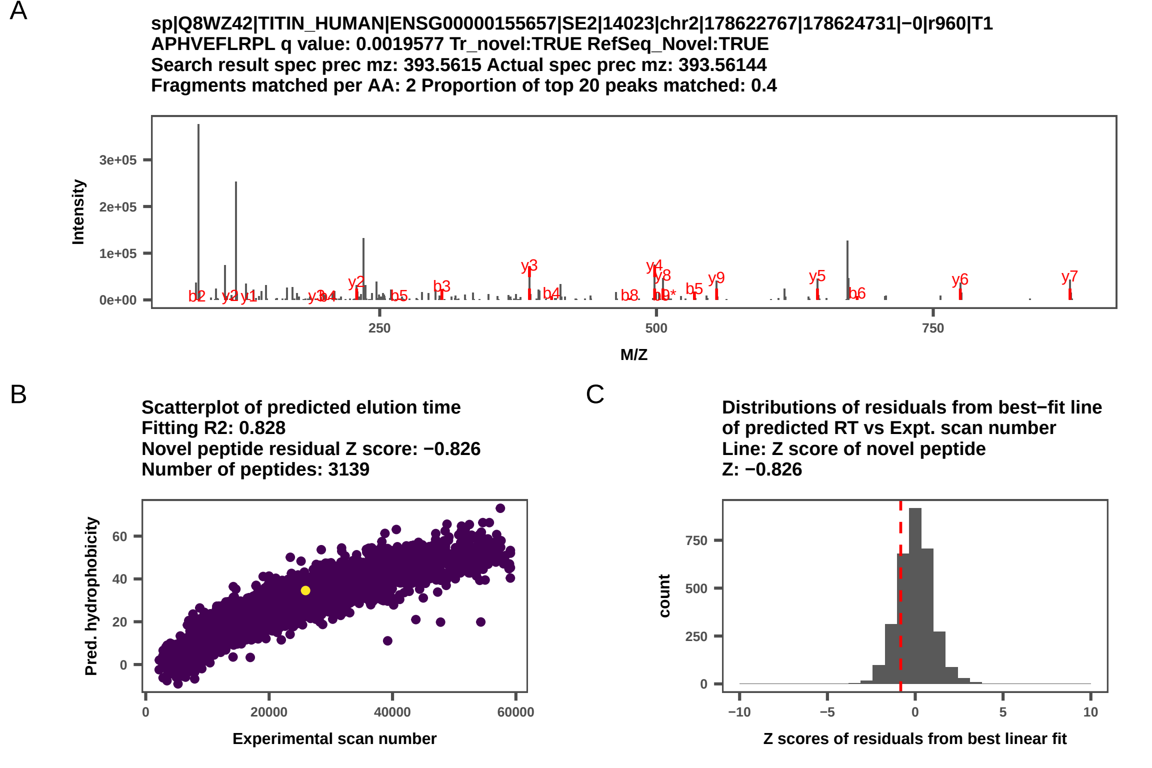
A
sp|Q8WZ42|TITIN_HUMAN|ENSG00000155657|SE2|14023|chr2|178622767|178624731|−0|r960|T1
APHVEFLRPL q value: 0.0019577 Tr_novel:TRUE RefSeq_Novel:TRUE
Search result spec prec mz: 393.5615 Actual spec prec mz: 393.56144
Fragments matched per AA: 2 Proportion of top 20 peaks matched: 0.4
Intensity
3e+05
2e+05
1e+05
0e+00
b2
y1
y2
b5
b3
y3
b4
b8
y4
y8
b5
y9
y5
b6
y6
y7
250
500
750
M/Z
B
Scatterplot of predicted elution time
Fitting R2: 0.828
Novel peptide residual Z score: −0.826
Number of peptides: 3139
Pred. hydrophobicity
60
40
20
0
0
20000
40000
60000
Experimental scan number
C
Distributions of residuals from best−fit line
of predicted RT vs Expt. scan number
Line: Z score of novel peptide
Z: −0.826
count
750
500
250
0
−10
−5
0
5
10
Z scores of residuals from best linear fit
